# A Systematic Review of Cutaneous Involvement in Metastatic Bone Sarcomas: Insights from 102 Reported Cases

**DOI:** 10.3390/cancers18030437

**Published:** 2026-01-29

**Authors:** Nikolaos Sideris, Efstratios Vakirlis, Elena Sotiriou

**Affiliations:** First Department of Dermatology and Venereology, Aristotle University of Thessaloniki, 54643 Thessaloniki, Greece

**Keywords:** bone sarcoma, cutaneous metastasis, osteosarcoma, chondrosarcoma, Ewing sarcoma, chordoma

## Abstract

**Background/Objectives:** Cutaneous metastases from primary bone sarcomas are exceedingly rare and poorly characterized, often posing diagnostic challenges due to their atypical presentation. This systematic review aimed to describe the clinical patterns, temporal relationships, and prognostic implications of cutaneous metastases across major bone sarcoma histologies. **Methods:** A comprehensive literature search was conducted to identify all reported cases of cutaneous metastases from osteosarcoma, chondrosarcoma, Ewing sarcoma, and chordoma. Data on patient demographics, primary tumor site, cutaneous lesion characteristics, latency periods, synchronous metastases, morphology, and clinical outcome were extracted and analyzed descriptively. **Results:** 102 cases were identified, with chordoma representing the most frequent histology. Cutaneous metastases showed histology-specific patterns: osteosarcoma and Ewing sarcoma typically presented with multiple lesions in the context of widespread systemic disease and poor prognosis, whereas chordoma more often exhibited solitary or skin-dominant metastases with longer latency and occasional favorable outcomes, including complete responses after local treatment. **Conclusions:** Cutaneous metastases in bone sarcomas display heterogeneous behavior, with chordoma demonstrating a more indolent and potentially manageable pattern compared to other histologies. Increased clinical awareness is essential to avoid diagnostic delays and optimize management.

## Simple Summary

Cutaneous metastases from primary bone sarcomas are very rare and not well understood, often leading to diagnostic delays or misinterpretation. This review analyzes over 100 reported cases to describe their clinical patterns, including location, timing, appearance, and outcomes across different sarcoma types. We found distinct behaviors: osteosarcoma and Ewing sarcoma typically show skin involvement as part of widespread disease with poor prognosis, while chordoma more often has skin-only or skin-dominant metastases with slower progression and occasional long-term survival. These findings highlight the need for greater awareness among clinicians to recognize unusual skin lesions in bone sarcoma patients, potentially enabling earlier diagnosis and better management.

## 1. Introduction

Bone sarcomas represent a heterogeneous group of rare malignant mesenchymal tumors that arise primarily in bone and are characterized by the production of osteoid, cartilage, or fibrous tissue. Among them, osteosarcoma, chondrosarcoma, chordoma and Ewing sarcoma constitute the most clinically relevant subtypes, with a predilection for children, adolescents, and young adults. Although their global incidence is low, bone sarcomas account for the majority of primary malignant bone tumors, and they are associated with aggressive biological behavior and significant morbidity and mortality.

The metastatic pattern of bone sarcomas is well established, with the lungs and, to a lesser extent, other bones being the most common secondary sites [1,2,3,4]. Cutaneous involvement, however, remains exceedingly rare. When it occurs, it not only signals advanced systemic disease but also poses diagnostic challenges due to its often non-specific clinical appearance. This is true not only for metastases from bone sarcomas but for most types of cancers [5,6,7]. For dermatologists, oncologists, and pathologists, awareness of this manifestation is crucial, as cutaneous metastases may occasionally represent the first sign of recurrence or disseminated disease.

This review aims to synthesize all available published cases of cutaneous metastases arising from osteosarcoma, chondrosarcoma, chordoma, Ewing sarcoma, and other rare bone sarcomas.

## 2. Clinicopathologic Overview

### 2.1. Clinical Presentation

In the literature, cutaneous metastases from bone sarcomas most often present as firm dermal or subcutaneous nodules, ranging from a few millimeters to several centimeters in diameter. Lesions are typically painless and may appear as solitary or multiple nodules. Less commonly, they may present as plaques, papules, ulcers, or inflammatory eruptions. Ulceration, hemorrhage, or rapid enlargement can occur in advanced cases [5,8]. The scalp, trunk, and proximal extremities are the most frequently reported anatomical sites, although virtually any skin region can be affected.

An important diagnostic challenge arises from their clinical resemblance to benign dermatologic conditions such as epidermal inclusion cysts, pyogenic granulomas, or vascular proliferations. Without a high index of suspicion, diagnosis may be delayed until histopathological confirmation is obtained [9]. In most reports, cutaneous involvement develops several months to years after the initial diagnosis of the primary sarcoma; however, rare cases have described cutaneous metastases as the presenting manifestation of previously undetected bone sarcoma.

Rapid growth, firm consistency, and persistence should prompt consideration of metastatic disease, especially in patients with a known history of bone sarcoma. Multiple or bilateral lesions should never be dismissed, as their presence often correlates with aggressive tumor biology and systemic dissemination.

### 2.2. Latency and Patterns of Cutaneous Metastases

Cutaneous metastases from bone sarcomas exhibit variable latency and distribution patterns depending on the histological subtype. In osteosarcoma, skin involvement typically arises several months to a few years after the initial diagnosis, often in the context of established pulmonary or osseous metastases [4,10]. Ewing sarcoma displays a broader temporal spectrum, with cutaneous lesions reported from only a few months up to multiple years post-diagnosis, frequently reflecting underlying systemic disease [11,12]. Chondrosarcomas and chordomas generally present cutaneous metastases late in the disease course, particularly in dedifferentiated or aggressive subtypes, whereas rarer sarcomas such as malignant fibrous histiocytomas, or giant cell tumors of bone show extremely sporadic skin involvement.

Certain patterns, such as bilateral hand involvement in chondrosarcoma [13] or multiple nodules on the scalp in osteosarcoma [14,15], have been documented, highlighting the diversity of clinical presentations. Prior surgical interventions or radiotherapy can occasionally act as conduits for metastatic seeding, underscoring the importance of evaluating areas of prior treatment.

### 2.3. Histopathology and Immunohistochemistry

A detailed histopathological description of cutaneous metastases from bone sarcomas is beyond the scope of this review. We therefore highlight the principal features. Comprehensive histopathological and immunohistochemical analyses can be found in dedicated pathology texts and prior studies.

They generally reflect the histologic features of the primary tumor, although they may appear more poorly differentiated.

Osteosarcoma: Metastatic lesions show malignant osteoid deposition with pleomorphic osteoblastic cells, sometimes forming lace-like trabeculae. Mitotic activity can be brisk, and areas of necrosis are common.

Ewing Sarcoma: Metastases typically consist of sheets of uniform small round blue cells with scant cytoplasm, often PAS-positive due to glycogen content. Necrosis and apoptosis may be present.

Chondrosarcoma: Cutaneous metastases reveal lobulated hyaline cartilage with atypical chondrocytes in lacunae, occasionally with myxoid change. Higher-grade or dedifferentiated variants show increased cellularity, nuclear atypia, and mitotic figures.

Chordoma: Metastatic lesions are characterized by physaliphorous cells with vacuolated cytoplasm arranged in cords or lobules within a myxoid stroma. Nuclear pleomorphism is usually mild, and mitotic figures are infrequent.

For extremely rare bone sarcomas, there are very few documented cutaneous metastases. Due to the scarcity of cases, the histology of cutaneous metastases is assumed to resemble that of the primary tumor, until further evidence becomes available from individual case reports. These cases should be evaluated carefully when new data are published, to confirm or refine this assumption.

### 2.4. Dermoscopy

Given the extreme rarity of cutaneous metastases from bone sarcomas, no studies have systematically evaluated their dermoscopic features. Available evidence derives mainly from broader studies on cutaneous metastases of mixed primary origin. A recently published multi-centre study [16] by the International Dermoscopy Society represents the largest series to date describing dermoscopic features of cutaneous metastases. 632 histologically confirmed lesions in 583 patients were included, although not a single patient had a primary bone sarcoma.

Most non-melanoma metastases were non-pigmented (pigmentation strongly suggests melanoma or breast primary) with prominent vascular features. Linear serpentine vessels were the most common vessel type. The structureless white pattern dominated overall. The recurring combination of structureless white areas with linear serpentine vessels, while non-specific, may serve as a valuable diagnostic clue to differentiate cutaneous metastases from other skin lesions or at least prompt biopsy in clinically ambiguous lesions. At present, however, dermoscopy cannot reliably distinguish metastatic sarcoma lesions from other malignant or benign cutaneous tumors and should be regarded as an adjunctive tool rather than a diagnostic modality.

### 2.5. Differential Diagnosis

Cutaneous nodules in patients with a history of bone sarcoma require careful evaluation, as they may mimic a wide spectrum of benign and malignant lesions. The differential diagnoses appear in Table 1:

## 3. Methods

### 3.1. Rationale for the Review

Despite increased recognition of cutaneous metastases as a marker of advanced disease, comprehensive data on their incidence, clinical behavior, and prognostic implications across bone sarcoma subtypes remain fragmented. Previous reviews have focused predominantly on single histologies or limited case series, precluding meaningful comparative analysis. To address this gap, we conducted a systematic review of all published cases of cutaneous metastases from primary bone sarcomas, encompassing osteosarcoma, chondrosarcoma, Ewing sarcoma, and chordoma. This review does not evaluate the overall metastatic distribution of primary bone sarcomas, but rather characterizes the clinicopathologic features of bone sarcomas after the development of cutaneous metastases. By aggregating individual patient-level data from 102 cases spanning 100 years, we aimed to delineate subtype-specific patterns of cutaneous involvement, temporal relationships, concomitant metastatic sites, and survival outcomes. The following sections present the largest and most detailed analysis of this rare phenomenon to date.

### 3.2. Literature Search and Record Screening

This systematic review was conducted in accordance with the Preferred Reporting Items for Systematic Reviews and Meta-Analyses (PRISMA) 2020 guidelines.

The study details and methodology were registered in the Open Science Framework (OSF) (https://osf.io/avkr9, (accessed on 3 January 2026)). To facilitate a transparent peer-review process, the registration is currently under embargo.

The systematic literature search across PubMed/MEDLINE, Google Scholar, and ScienceDirect yielded a total of 5673 records after duplicate removal. The search strategy used the following core terms: (“osteosarcoma” OR “osteogenic sarcoma” OR “chondrosarcoma” OR “Ewing sarcoma” OR “Ewing’s sarcoma” OR “chordoma” OR “primary bone sarcoma” OR “skeletal sarcoma”) AND (“cutaneous metastasis” OR “cutaneous metastases” OR “skin metastasis” OR “skin metastases” OR “subcutaneous metastasis” OR “cutaneous involvement” OR “skin involvement” OR “cutaneous deposit” OR “metastatic skin lesion” OR “skin secondary” OR “dermal secondary”). Minor variations were applied to optimize retrieval in each database. The search was conducted from database inception to 31 October 2025.

Following title and abstract screening, 5562 records were excluded, and 111 reports underwent full-text assessment. Of these, 23 reports could not be retrieved as full text: 5 were completely inaccessible and thus excluded, while 18 provided sufficient extractable partial data from abstracts, PubMed summaries, or secondary sources (mainly prior reviews citing these studies). These partial data included key variables such as patient demographics, primary tumor site and histology, cutaneous lesion characteristics, latency periods, concomitant metastatic sites, morphology, and clinical outcome, allowing their inclusion without compromising the integrity of the analysis.

**Figure 1:**
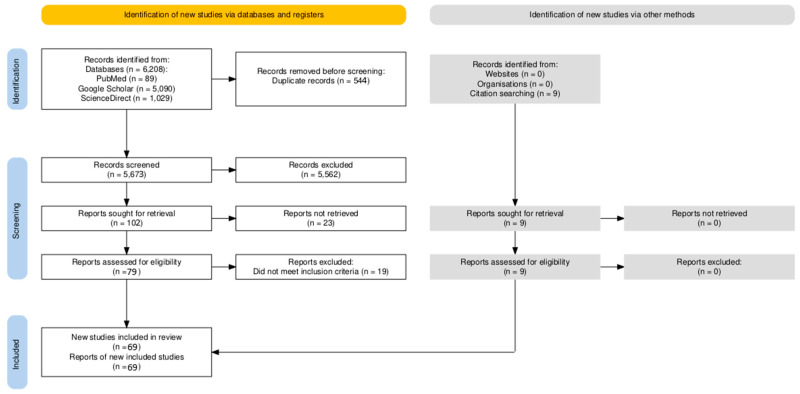
PRISMA flow diagram of study selection.

The remaining 88 full-text articles were evaluated, of which 69 met the inclusion criteria. In total 87 reports were included in the review [4,10,11,12,13,14,15,17,18,19,20,21,22,23,24,25,26,27,28,29,30,31,32,33,34,35,36,37,38,39,40,41,42,43,44,45,46,47,48,49,50,51,52,53,54,55,56,57,58,59,60,61,62,63,64,65,66,67,68,69,70,71,72,73,74,75,76,77,78,79,80,81,82,83,84,85,86,87,88,89,90,91,92,93,94,95,96] 69 with complete full-text access and 18 with partial data from alternative sources) (Figure 1). The included cases spanned publications from 1924 to 2024, with the majority (53/87, 61%) reported after 2000. Search was limited to English, French, and German languages. Historical cases (pre-1950) reflect evolving classifications and were included only if descriptions aligned with contemporary histologic standards, ensuring relevance and compatibility.

Two independent reviewers (SN and VE) conducted the screening and eligibility assessment, including title/abstract screening and full-text evaluation. Discrepancies were resolved by consensus or, when necessary, by consultation with a third reviewer (SE). Data extraction was performed using a standardized spreadsheet (Microsoft Excel) by one reviewer (SN) and verified by a second (VE). The extracted data elements were categorized into:

- Study and patient characteristics: First author, year of publication, DOI/link, study design, patient age, and sex.
- Primary tumor data: Specific histological subtype and anatomical site of the primary bone sarcoma.
- Cutaneous metastasis details: Anatomical location, clinical morphology (e.g., nodule, plaque), distribution (solitary vs. multiple), and the latency period between primary diagnosis and skin involvement.
- Diagnostic and treatment Information: Diagnostic methods (biopsy, FNAC), key immunohistochemical (IHC) markers, and local or systemic therapeutic interventions.
- Clinical Outcomes: Presence of other metastatic sites, overall survival from the time of cutaneous metastasis, and patient status.

The standardized spreadsheet with all collected data (Supplementary Materials File S1) can be found in the Supplementary Material section of the article. Data were analyzed using descriptive statistics. Categorical variables were expressed as frequencies and percentages, while continuous variables (e.g., age, latency, survival) were summarized using means, medians, and ranges.

No standardized risk-of-bias assessment tool was applied to the included studies, as validated tools are not designed for case reports or case series.

### 3.3. Inclusion and Exclusion Criteria

The inclusion criteria for articles were:

- Histologically confirmed primary bone sarcoma (osteosarcoma, chondrosarcoma, Ewing sarcoma, chordoma). One additional case of cutaneous metastasis from an ultra-rare bone sarcoma was identified but excluded from the primary analysis because of the small number of cases: a scalp metastasis from malignant fibrous histiocytoma of bone [97], an entity whose name is now replaced by undifferentiated pleomorphic sarcoma.
- Documented cutaneous or subcutaneous metastasis.
- Case reports, case series, cohort studies with individual patient data.

The exclusion criteria were:

- Bone sarcomas without cutaneous metastases.
- Non-sarcoma bone tumors.
- Other sarcomas.
- Language other than English, French, or German.
- Duplicate or overlapping reports.
- Eligible sarcomas (osteosarcoma, Ewing sarcoma, chordoma, or chondrosarcoma) that arose from extra-osseous sites.

## 4. Results

**Figure 2:**
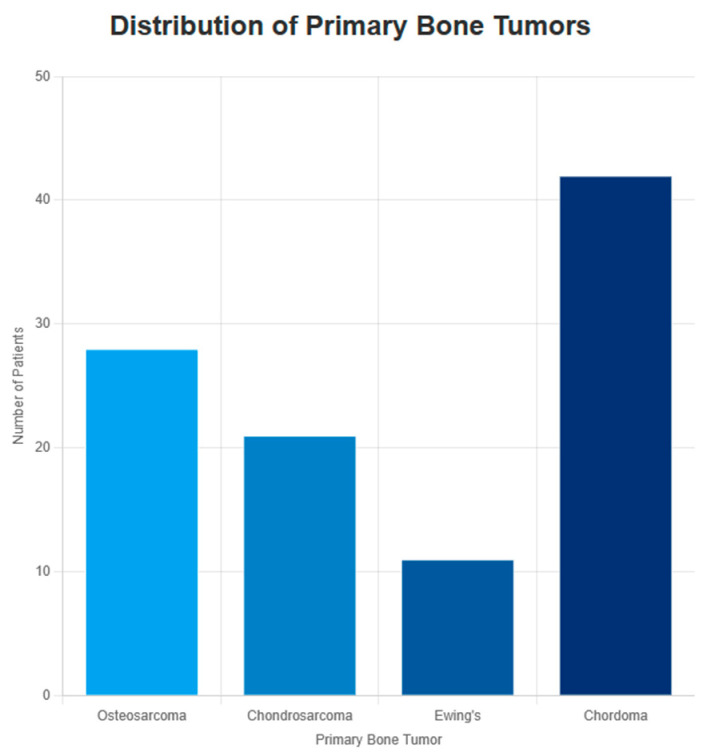
Distribution of primary bone tumors in the 102 patients included in the review.

The final cohort comprised 102 patients with cutaneous metastases from primary bone sarcomas: 28 patients with osteosarcoma, 21 with chondrosarcoma, 11 with Ewing sarcoma, and 42 with chordoma (Figure 2). The individual cases included in this analysis are detailed in the Supplementary Materials, allowing interested readers to review the underlying patient-level data.

**Figure 3:**
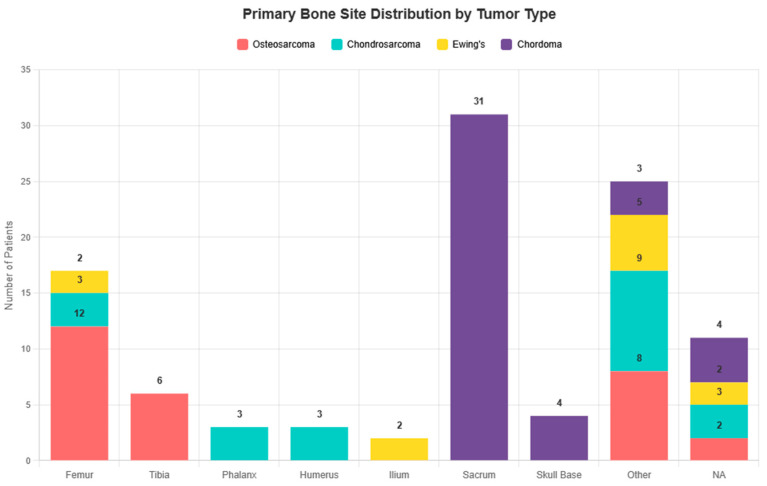
Primary bone site distribution by tumor type.

The distribution of primary bone tumor sites varied markedly by histology. The femur was the most common site in osteosarcoma, the sacrum in chordoma, and long bones in Ewing sarcoma and chondrosarcoma (Figure 3).

Median age at diagnosis of cutaneous metastasis was 47 years (IQR 23–61, range 2–88, n = 85/102—age was not reported in 17 cases). Age distribution varied markedly across histologies, with Ewing sarcoma (median age: 18 years (IQR 13–23, range 7–40, n = 10) and osteosarcoma (median age: 23 years (IQR 16–46, range 12–75, n = 23) affecting predominantly younger patients, whereas chondrosarcoma (median age: 44 years (IQR 36–59, range 30–85, n = 17) and chordoma (median age: 60 years (IQR 47–64, range 2–88, n = 35) occurred in older individuals.

Male predominance was observed overall (59%, n = 50/85). Sex distribution was relatively balanced in all types of tumors (osteosarcoma: 10 m/13 f, chondrosarcoma: 10m/7f, Ewing sarcoma 5m/5f) while chordoma was more frequent in males (25m/10f). In 17 cases, sex of the patient was unknown.

**Figure 4:**
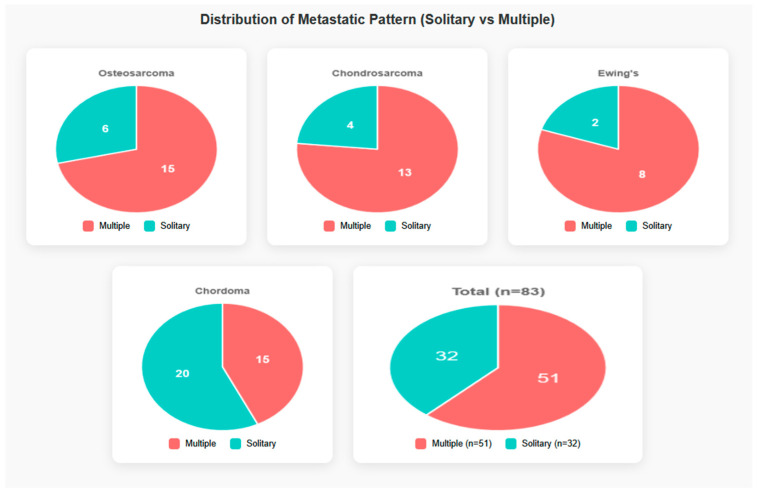
The distribution of metastatic pattern (solitary vs. multiple) by bone tumor type. Only cases with reported pattern are included—NA excluded from visualization.

**Figure 5:**
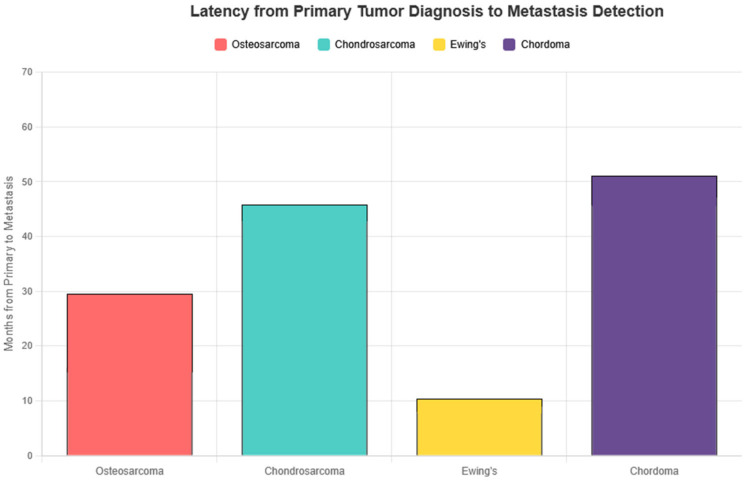
Mean latency (months) from primary tumor diagnosis to cutaneous metastasis detection. Only numeric values were included. NA values were excluded. “Preceded detection of main tumor” and “few days” were coded as 0 months.

Cutaneous metastatic lesions were multiple in the majority of cases (61%) and solitary in the remainder (Figure 4). Synchronous presentation (cutaneous metastasis at or within one month of primary diagnosis) was uncommon and occurred mainly in Ewing sarcoma and in some cases of osteosarcoma. Latency from primary bone sarcoma diagnosis to cutaneous metastasis varied significantly among subtypes, ranging from a median of 1.5 months in Ewing sarcoma to 48 months in chordoma (Figure 5). In detail, latency (in months) from primary bone sarcoma diagnosis to cutaneous metastasis was as follows: osteosarcoma: mean 29.5, median 9.5; chondrosarcoma: mean 45.3, median 24; Ewing sarcoma: mean 10.6, median 1.5; chordoma: mean 51.4, median 48.

The most common sites of cutaneous metastases overall were the trunk (29%), scalp (20%), and extremities (18%), followed by the face (12%) and head/neck region (11%). Distinct anatomical preferences emerged by histology: osteosarcoma frequently involved the scalp (46% of cases), chondrosarcoma showed a striking predilection for acral sites (especially hands), chordoma most commonly affected the trunk (43%) and face (26%), while Ewing sarcoma predominantly involved the trunk (55%).

A total of 136 cutaneous metastases sites were recorded across 102 patients, with multiple sites per patient counted individually. Data considering the sites of metastases are presented in Table 2.

As for other sites of metastatic disease, cutaneous involvement most commonly occurred in the context of established systemic spread, with pulmonary metastases representing by far the predominant concomitant distant site. Analytically, for patients for whom data were available:

- Osteosarcoma (n = 22): 17 had pulmonary metastases (either alone or concomitant with other sites), 3 had metastases at other sites, and in only 2 of them metastatic disease was limited to the skin.
- Chondrosarcoma (n = 16): 15 had pulmonary metastases, only 1 had metastases limited to the skin.
- Ewing sarcoma (n = 7): 3 had pulmonary metastases, and 4 had metastases at other sites.
- Chordoma (n = 27): 13 had pulmonary metastases, 9 had metastases at other sites, and 5 had metastases limited to the skin.

**Figure 6:**
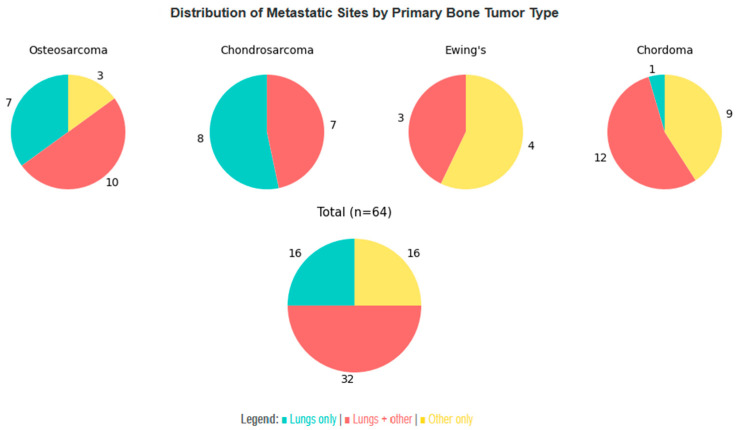
Distribution of concomitant metastatic sites in patients with cutaneous metastatic disease. Patients with no data available or without metastases outside the skin are not included in the visualization.

Sites of metastatic disease other than the lungs were most commonly other bones, brain, lymph nodes, and liver. The strong predilection of bone sarcomas to metastasize to the lungs is illustrated in Figure 6. Key observations from the figure and our data are:

- Patients with osteosarcoma and chondrosarcoma who have metastatic disease concomitant with the skin have an 85% and 100% probability, respectively, of pulmonary involvement, either alone or together with other sites.
- Although the sample size is small, Ewing sarcoma appears to exhibit a broader and more heterogeneous pattern of metastatic spread.
- It is very rare for chordoma patients to have metastases limited to the skin and lungs; they usually involve the lungs together with other sites, or other sites without lung involvement.

**Figure 7:**
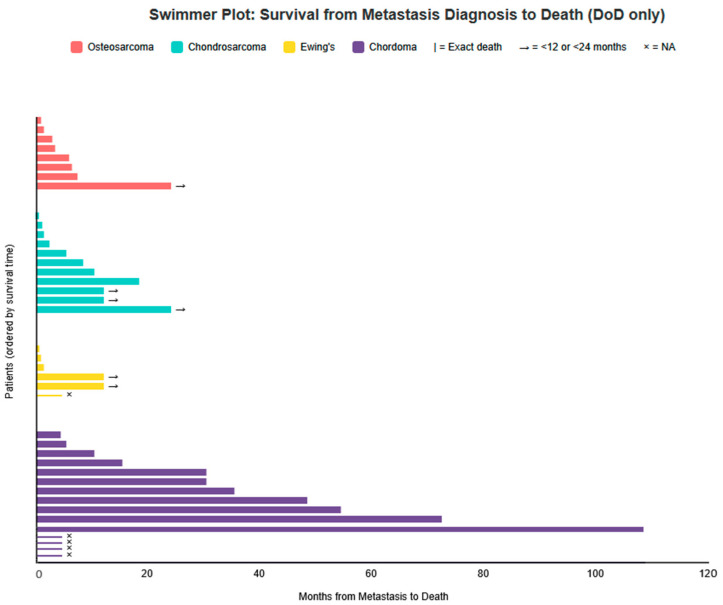
Swimmer plot showing survival time (months) from metastasis diagnosis to death for patients with Died of Disease (DoD) outcome. Each bar represents one patient. Patients are ordered from shortest to longest survival within each tumor type. Osteosarcoma (n = 8): 0.5, 1, 2.5, 3, 5.5, 6, 7, <24 months. Chondrosarcoma (n = 11): 0.1, 0.7, 1, 2, 5, 8, 10, 18, <12, <12, <24 months. Ewing sarcoma (n = 6): 0.2, 0.5, 1, <12, <12 months and one NA. Chordoma (n = 15): 4, 5, 10, 15, 30, 30, 35, 48, 54, 72, 108 months and four NA.

Of the 102 patients with metastatic disease, clinical outcome was known in 62 cases. Forty patients (65% of known) died of disease (DoD), including three chordoma patients with documented severe and progressive metastatic disease who were classified as DoD. Nineteen patients (31%) were alive with disease (AwD) at last follow-up, and three (5%) achieved no evidence of disease (NED)/complete response after treatment of metastases (one osteosarcoma and two chordoma). Prognosis varied substantially by histology: Ewing sarcoma showed the highest DoD rates (6/7 known cases). Chordoma had the most favorable outcome, with 11 AwD and 2 NED among 28 known cases (Table 3). Individual post-metastasis survival trajectories for DoD patients are illustrated in the swimmer plot (Figure 7).

The swimmer plot (Figure 7) should be interpreted with caution, as it illustrates individual survival times for patients who DoD. It is important to note that this figure includes only deceased patients and therefore depicts the worst-case scenarios; the 19 patients AwD and 3 in complete remission are not shown, resulting in a more favorable overall prognosis than suggested by the plot alone. Conversely, AwD patients have censored follow-up and may die soon after last contact or survive for long time, precluding overestimation of favorable prognosis from censored data alone.

## 5. Discussion

Cutaneous metastases from primary bone sarcomas represent an exceptionally rare manifestation of systemic disease. While the metastatic behavior of bone sarcomas has been extensively studied with respect to pulmonary and skeletal dissemination, skin involvement has remained largely confined to isolated case reports and small series. By aggregating all available published cases over a period of approximately one century, the present study provides the most comprehensive overview to date of cutaneous metastases arising from osteosarcoma, chondrosarcoma, Ewing sarcoma, and chordoma, allowing meaningful comparisons across histologic subtypes.

Our analysis confirms that cutaneous metastases generally occur in the setting of advanced disease, most often alongside pulmonary and/or other distant metastases. However, important subtype-specific differences emerge with respect to latency, anatomical distribution, and prognosis. These findings suggest that cutaneous involvement is not a uniform phenomenon across bone sarcomas, but rather reflects distinct biological behaviors inherent to each tumor type.

Latency from primary tumor diagnosis to detection of cutaneous metastasis varied markedly between subtypes. Ewing sarcoma demonstrated the shortest interval, with a median latency of only 1.5 months, often presenting synchronously or shortly after initial diagnosis. This rapid dissemination is consistent with the known aggressive biology and early metastatic potential of Ewing sarcoma. In contrast, chordoma exhibited a strikingly prolonged latency, with a median of 48 months, underscoring its characteristically indolent yet persistent course. Osteosarcoma and chondrosarcoma showed intermediate latency patterns, though with wide variability. These temporal differences are clinically relevant, as they may inform surveillance strategies and diagnostic suspicion at different stages of disease.

Anatomical distribution of cutaneous metastases also differed substantially by histologic subtype. Osteosarcoma showed a notable predilection for the scalp, accounting for nearly half of reported cases, a pattern that has been sporadically noted in the literature but not previously quantified at scale. Chondrosarcoma demonstrated a striking tendency toward acral involvement, particularly of the hands, while chordoma most frequently affected the trunk and face. Ewing sarcoma predominantly involved the trunk. The mechanisms underlying these site-specific patterns remain speculative. Possible contributing factors include regional vascularity, venous drainage pathways, prior surgical or radiotherapy fields, and tissue-specific microenvironmental factors that may facilitate tumor cell seeding and growth. Reporting bias cannot be excluded, particularly for highly visible sites such as the scalp or face; nevertheless, the consistency of these patterns across multiple decades suggests that non-random biological factors may be involved.

From a prognostic standpoint, the presence of cutaneous metastases generally signaled advanced disease and poor outcome, particularly in osteosarcoma, chondrosarcoma, and Ewing sarcoma, where the majority of patients with known outcomes died of disease within months of cutaneous involvement. Ewing sarcoma, in particular, was associated with the highest mortality rate following cutaneous metastasis, albeit based on a limited number of cases. In contrast, chordoma again emerged as an outlier: a substantial proportion of patients were alive with disease at last follow-up, and rare cases achieved complete remission after treatment of metastatic lesions. These findings caution against viewing cutaneous metastases as uniformly terminal events and highlight the importance of histologic context when counseling patients and planning management.

Clinically, cutaneous metastases from bone sarcomas often present as firm, painless dermal or subcutaneous nodules that can closely mimic benign conditions such as epidermal inclusion cysts, vascular proliferations, or inflammatory lesions. In several reports, diagnosis was delayed due to their non-specific appearance. Awareness of this rare but significant manifestation is therefore essential, particularly for dermatologists evaluating new or rapidly growing skin lesions in patients with a current or prior history of bone sarcoma. Prompt biopsy and histopathologic correlation remain crucial, as early recognition may lead to timely restaging and therapeutic intervention. Notably, in our cohort, the diagnosis of cutaneous metastasis was established overwhelmingly by skin biopsy, underscoring its central role in confirming metastatic disease in this setting.

Representative clinical images could not be included in the present review, as the available literature consists almost exclusively of isolated case reports with heterogeneous documentation, and no standardized image sets were retrievable despite direct inquiries to dermatology and pathology departments in multiple institutions, both in our and other countries. As a result, no uniform or truly representative photographic material could be assembled without introducing selection bias.

The present study has several limitations inherent to its design. Clinical and follow-up data were incomplete in a substantial proportion of cases, precluding robust survival analyses and multivariable modeling. Additionally, treatment modalities for both primary tumors and metastatic disease varied widely across eras and institutions, limiting conclusions regarding therapeutic impact. Partial data extraction from abstracts or secondary sources was performed for 18 reports where full texts were unavailable. Although this may introduce minor information bias for variables not uniformly reported across all sources, only clearly and explicitly stated information from these sources was included in the analysis, ensuring the robustness of the main subtype-specific patterns observed.

Furthermore, the reliance on published case reports and small series inevitably introduces reporting and publication bias. Unusual or dramatic cases (such as solitary lesions with prolonged survival or atypical anatomical presentations) may be overrepresented, as they are more likely to be submitted for publication. Conversely, clinically silent, incidental, or cutaneous metastases not leading to notable outcomes are likely underreported, potentially skewing our dataset toward more severe or noteworthy manifestations.

This underscores the need for cautious interpretation of our findings and highlights the potential value of future multicenter registries designed to capture a broader and less biased spectrum of cutaneous involvement in bone sarcomas.

Despite these limitations, the aggregation of 102 individual cases allows for patterns to emerge that would not be apparent from isolated reports.

In conclusion, cutaneous metastases from primary bone sarcomas are rare but clinically meaningful events that exhibit distinct patterns according to histologic subtype. Differences in latency, anatomical distribution, and outcome underscore the heterogeneous biology of these tumors and challenge the notion of cutaneous involvement as a uniform marker of terminal disease. Increased awareness of these patterns may facilitate earlier diagnosis, appropriate staging, and more nuanced prognostic assessment in affected patients. Future multicenter registries and collaborative efforts may further clarify the biological mechanisms and optimal management of this unusual manifestation of bone sarcoma dissemination.

## 6. Conclusions

Clinicians, particularly dermatologists and oncologists, should maintain a high index of suspicion for cutaneous metastases in patients with a history of primary bone sarcoma who present with new, firm, painless dermal or subcutaneous nodules, especially when arising in histology-specific predilection sites (e.g., scalp in osteosarcoma, acral areas in chondrosarcoma, trunk and face in chordoma). Rapid growth, multiplicity, or lesion persistence should prompt immediate evaluation. Biopsy remains the cornerstone of diagnosis, as these lesions frequently mimic benign conditions, and early histologic confirmation can facilitate timely restaging and appropriate management.

Prognostic implications vary by histologic subtype: cutaneous involvement in osteosarcoma, chondrosarcoma, and Ewing sarcoma typically reflects advanced systemic disease with poor outcomes, often associated with survival measured in months. In contrast, cutaneous metastases in chordoma may reflect a more indolent disease course, with potential for prolonged survival or, in selected cases, meaningful responses to local therapies. These differences should be considered during patient counseling and individualized treatment planning.

## Figures and Tables

**Table 1:** Differential diagnoses of cutaneous nodules in patients with bone sarcomas.

Category	Examples
Benign cutaneous conditions	Epidermal inclusion cyst, Pilar cyst, Dermatofibroma, Lipoma, Pyogenic granuloma, Vascular malformation (hemangioma)
Primary cutaneous malignancies	Basal cell carcinoma, Squamous cell carcinoma, Melanoma, Cutaneous adnexal tumor
Secondary/metastatic lesions (non-sarcoma primaries)	Carcinomas of breast, lung, colon, kidney, thyroid; Lymphoma or leukemia cutis; Other soft tissue sarcomas (e.g., GIST)
Infectious or inflammatory mimics	Abscess, Cellulitis, Nodular panniculitis, Granulomatous diseases (sarcoidosis, foreign body reaction)

**Table 2:** Distribution of cutaneous metastasis sites by histologic subtype (n = 102 cases/136 sites).

Subtype	Scalp	Head/Neck	Trunk	Extremity	Foot	Hand	Face	Other
Osteosarcoma (n = 28)	13	5	7	5	1	0	0	0
Chondrosarcoma (n = 21)	5	1	9	5	1	8	4	2
Ewing Sarcoma (n = 11)	1	1	6	3	0	0	1	2
Chordoma (n = 42)	8	8	18	11	0	0	11	0

**Table 3:** Clinical outcomes of all patients included in the review. DoD = died of disease, AwD = alive with disease, NED = no evidence of disease, NA = data not available.

Tumor	DoD	AwD	NED	NA	Total
Osteosarcoma	8	4	1	15	28
Chondrosarcoma	11	3	0	7	21
Ewing sarcoma	6	1	0	4	11
Chordoma	15	11	2	14	42
Total	40	19	3	40	102

## Data Availability

The raw data supporting the conclusions of this article are provided as Supplementary Materials File S1, containing the complete case-by-case dataset extracted from all included reports.

## Supplementary Materials

The following supporting information can be downloaded at: https://www.mdpi.com/article/10.3390/cancers18030437/s1, File S1: Microsoft Excel spreadsheet containing the complete case-by-case dataset, including all extracted variables and references for the 102 included cases.

## Abbreviations

The following abbreviations are used in this manuscript:

DoD	died of disease
AwD	alive with disease
NED	no evidence of disease
NA	data not available
